# Multi-state occupancy models of foraging habitat use by the Hawaiian hoary bat (*Lasiurus cinereus semotus*)

**DOI:** 10.1371/journal.pone.0205150

**Published:** 2018-10-31

**Authors:** P. Marcos Gorresen, Kevin W. Brinck, Megan A. DeLisle, Kristina Montoya-Aiona, Corinna A. Pinzari, Frank J. Bonaccorso

**Affiliations:** 1 Hawai‘i Cooperative Studies Unit, University of Hawai‘i at Hilo, Hilo, Hawai‘i, United States of America; 2 U.S. Geological Survey, Pacific Island Ecosystems Research Center, Hawaii National Park, Hawai‘i, United States of America; University of Western Ontario, CANADA

## Abstract

Multi-state occupancy modeling can often improve assessments of habitat use and site quality when animal activity or behavior data are available. We examine the use of the approach for evaluating foraging habitat suitability of the endangered Hawaiian hoary bat (*Lasiurus cinereus semotus*) from classifications of site occupancy based on flight activity levels and feeding behavior. In addition, we used data from separate visual and auditory sources, namely thermal videography and acoustic (echolocation) detectors, jointly deployed at sample sites to compare the effectiveness of each method in the context of occupancy modeling. Video-derived observations demonstrated higher and more accurate estimates of the prevalence of high bat flight activity and feeding events than acoustic sampling methods. Elevated levels of acoustic activity by Hawaiian hoary bats were found to be related primarily to beetle biomass in this study. The approach may have a variety of applications in bat research, including inference about species-resource relationships, habitat quality and the extent to which species intensively use areas for activities such as foraging.

## Introduction

Monitoring bat populations is challenging because many species occur at low densities, are difficult to detect, and have wide-ranging movement and migratory patterns that are poorly understood [1]. Foliage-roosting “tree bats” (a group of about 17 species of Lasiurus and the silver-haired bat, *Lasionycteris noctivagans*) pose a particular challenge as populations are typically "over-dispersed" [2], and may be vocally cryptic or not readily accessible to acoustic sampling or capture [3–6]. Quantifying conventional population parameters such as abundance or density for these species is not currently feasible and can hamper species status and conservation assessments [1]. For such species, occupancy analysis can be an effective method for elucidating the relationship of habitat attributes and species occurrence while also accounting for imperfect detection [7], and the approach has been used for bat studies at local [8,9], regional [10] and broader geographic extents [11,12]. However, analyses based solely on animal presence can be inadequate because they omit information on abundance, activity levels, or behavior that may further inform assessment of habitat use and suitability. Advances in occupancy modeling have expanded its application to incorporate additional information about a range of biologically relevant states such as behavioral attributes (e.g., absent or not detected, present but possibly not reproducing, and present and reproducing; [13]) or categorical levels of activity (e.g., calling rates; [14,15]) and abundance (e.g., “none, some, many”; [16]). These “multi-state” models characterize occupancy as a hierarchical categorical variable, and accommodate uncertainty caused by imperfect detection in both the estimation of species occurrence and classification of the correct state.

Given their flexibility, multi-state occupancy models may serve to evaluate the relative use or quality of areas used by bats for foraging, roosting, or other requirements. Bat habitat suitability can be assessed in a number of ways, including for example, studies of focal use of areas by radio-tracked individuals [17], body weight [18], reproductive condition [19], and dietary richness [20]. However, these approaches typically require bat capture and handling which can be difficult or impractical in many settings. Non-invasive observations of bat detection rates and feeding behavior may provide alternate metrics that, coupled with multi-state occupancy analysis, reveal the degree to which an area is used for foraging by a resident population and the habitat attributes associated with state variables of interest (e.g., high abundance or activity), information of relevance to land managers and conservation efforts.

Optimal foraging theory posits that fitness is related to foraging efficiency, and that the rate at which energy is obtained is partly a function of time spent in a particular habitat [21,22]. Insectivorous bats expend considerable time and energy foraging (e.g., more than half the energy budget; [23]), with individuals often consuming over a quarter of their body weight in invertebrates each night, particularly during energetically demanding periods [24]. Thus, bat occurrence and activity are expected to be generally associated with insect abundance, a prediction broadly supported by various studies (e.g., [25–27]), although this relationship can be temporally variable and depend on resource availability and prey selection [28,29]. Monitoring insectivorous bat distribution and habitat use is a particularly pressing issue given observed declines in many insectivorous bird populations (e.g., [30]), a pattern that may be linked to diminished flying insect populations and ecosystem changes [31,32].

The primary objective of our study is to investigate the use of multi-state occupancy modeling to quantify foraging habitat use and suitability by Hawaiian hoary bats (*Lasiurus cinereus semotus*, Vespertilionidae), and constitutes the first application of the method to observations of bat activity and behavior. Also known as the ‘Ōpe‘ape‘a, the species is the only extant native terrestrial mammal and sole bat species in Hawai‘i state and occurs on all of the major islands [33]. It is an aerial-hawking bat that feeds primarily on Coleoptera and Lepidoptera captured and eaten while in flight [34–37]. The Hawaiian hoary bat served as the focal species in this study because it is an endangered endemic susceptible to fatality by collision with wind turbine blades [38] and the subject of management aimed at mitigating these impacts [39]. The North American subspecies, *L*. *c*. *cinereus*, also accounts for approximately 40% of all bat fatalities at turbines in continental North America [40]. We specifically examine the issue of foraging habitat use because land-cover restoration is a conservation approach applied to offset Hawaiian hoary bat fatalities and requires an understanding of current bat activity before management efforts begin and from which to compare results.

We applied the multi-state occupancy approach to analyze two distinct types of behavioral data amenable to classification as variable “states”—flight activity levels and feeding behavior. Survey methods that rely on acoustic sampling are widely used to determine species presence and relative activity, but bats may at times be vocally cryptic and remain undetected [5,6]. Therefore, we used data derived from separate visual and auditory sources, namely thermal videography and acoustic (echolocation) detectors, jointly deployed at each sample site to secondarily compare how sampling techniques affect habitat use inferences. We speculate that prey availability may be a limiting resource regulating Hawaiian hoary bat occurrence and activity; as such, we focus exclusively on insect biomass as a predictive variable of bat occupancy and habitat use. This focus also helped ensure that models were not over-parameterized and were able to accommodate our limited sample size. We discuss the use of multi-state occupancy models to draw inference about habitat suitability and the assumptions relevant to applying such models to local populations comprised of wide-ranging individuals.

## Methods

### Study area and data collection

The field study was conducted from 10 July to 10 August 2017 in the northern Ko‘olau Mountains of O‘ahu, within a 25 sq km area managed for wind energy production (centered at 21°36'21"N, 158°2'14"W). The region is comprised of a mix of active and fallow cattle pasture and forest, with the latter dominated by introduced species at lower elevations and native species at higher elevations (generally >300 m). Topography consists of incised hillsides with elevation at sample sites ranging from 145 to 360 m above sea level. Four sites, located an average of 2.5 km apart, were concurrently sampled four nights each week for a period of five weeks (20 sites total). Candidate sites based primarily on a road and trail network were obtained from a generalized random-tessellation stratified (GRTS) sampling design produced with the R package spsurvey version 3.3 [41]. Stratification was applied by dividing the study area into quadrants, and sites were selected from the candidate pool given the constraint of a 1-km minimum spacing between sites sampled concurrently. The selection of the study area for sampling and testing multi-state occupancy models was based on a previous study demonstrating bat occurrence in the region [38].

Bat activity and behavior was quantified both visually and acoustically. Visual detection of bats was achieved using surveillance cameras equipped with 19-mm lenses (Axis Q1922-E, Axis Communications, Lund, Sweden). These cameras image in the “thermal” spectrum of infrared light (approximately 9,000–14,000 nm) and require no supplemental illumination. At each sample site, a camera was set in an open area and aimed upward at a 45° angle so as to exclude any nearby vegetation. Previous trials showed that bats were detectable at distances of over 100 m. Video imagery was processed using custom-written code and matrix-based statistical software (Mathworks, Natick, Massachusetts, USA) to automatically detect animals flying through the video scenes. Video was recorded at 30 frames per second, and every 10th video frame was analyzed resulting in the detection of events lasting as little as 0.3 sec. All objects detected by software algorithms were visually reviewed and identified as bat, bird or insect. Bat detections occurring ≤1 minute apart were counted as a single event, and counts of these events by night and site were used to measure relative activity. Although use of a 1-minute threshold was arbitrary, about 84% of video detections of bat flight were comprised of single passes, and the time difference between all detection events averaged 21 minutes, reflecting the occasionally clustered but generally sparse distribution of the visual observations. In addition to the video-based index of activity, observations of flight trajectories that included a rapid loop or roll (accomplished in ≤1 s) were used to indicate active prey targeting. Aerial-hawking species such as *L*. *borealis* and *L*. *cinereus* that have a moderate to high wing-load (ratio of mass to wing area) and aspect ratio (wingspan^2^/wing area) are fast and agile fliers with the ability to rapidly initiate a roll and alter their flight path while in pursuit of prey [42–44]. Nightly counts of these observations were used to quantify the number of feeding attempts at each site.

Bat echolocation was acoustically monitored with ultrasonic detectors (Song Meter 4 Bat FS, Wildlife Acoustics, Inc., Concord, Massachusetts, USA), each equipped with a directional horn-mounted SMM-U1 microphone oriented towards the air-space imaged by a video camera. Detectors began recording 30 minutes before local sunset until 30 minutes after sunrise the next morning. Acoustic events were recorded without digital compression as full-spectrum wav sound files with the following settings: sampling rate of 192 kHz; high pass filter at 16 kHz and 12 decibel gain; microphone bias off; digital high pass filter at fs/24; digital low pass filter off; trigger level 6 decibel signal-noise ratio; trigger window 3.0 sec; trigger max length 15 sec; frequency division ratio 16. Kaleidoscope Pro (version 4.1.0a; Wildlife Acoustics, Inc.) software was used to review files and filter acoustic background noise with the following settings: signal of interest between 8 and 80 kHz, 2 to 250 ms pulse duration, and a minimum of 2 pulses per event. All files classified as containing bat echolocation pulses were visually and aurally inspected as sonograms with Kaleidoscope Pro (version 3.1.0; Wildlife Acoustics) to ensure that there were no false positives. As with video detections, acoustic detections occurring ≤1 minute apart were counted as a single event, and counts of these events by night and site were used as an acoustic-based index of activity. Terminal-phase calls (“feeding buzzes” emitted just prior to an attempted insect catch [45]) were qualitatively distinguished from search and approach-phase calls by a rapid increase in the call rate. In addition to the acoustic index of activity, counts of terminal-phase call events were used to quantify feeding events per night.

To determine the abundance of potential bat prey, insects were sampled at each site with an ultraviolet fluorescent light trap (Leptraps, Georgetown, Kentucky, USA). Trapped insects were collected following each night of sampling, sorted to size and order for insects with a body length ≥5 mm, and oven-dried for 48 h at 65°C. Data used for analyses were restricted to biomass (dry weight) tallies of Coleoptera and Lepidoptera in the size class ≥5 to 20 mm (summarized in S1 Table). Moths and beetles with body lengths >20 mm made up 15% of all insect weight but comprised only 1% of counts and were generally larger than 24 mm, the maximum length of prey items consumed by Hawaiian hoary bats [36]. These captures were excluded from analyses to minimize the effect of outliers.

All sampling protocols for this study were approved by IACUC at the University of Hawai‘i at Hilo. Access to the wind power facility was granted by D.E. Shaw Renewable Investments IV, L.L.C.

### Occupancy modeling

We used two types of multi-state models to investigate foraging habitat use by bats. The first model applied flight activity categories derived from nightly tallies of bat detections (“activity” model), and the second model used feeding attempts by bats evident from flight trajectories and terminal-phase vocalizations (“feeding” model). Both of these models were developed separately for the data obtained from the two different survey methods (“acoustic”, “video”). Altogether the analyses consist of four model types: Acoustic–activity, Acoustic–feeding, Video–activity, and Video–feeding.

Each of the models produced estimates of the probability of observing the species in state 1 given its true state was 1 (*p*^1^), and the probability of observing the species in state 2 given its true state was 2 (*p*^2^). Put another way, for the activity model these are the probabilities of observing at least one bat at sites at which activity was truly low (*p*^1^) or high (*p*^2^), respectively. For the feeding model, they are the probabilities of observing at least one bat at sites where feeding was not occurring or was actually occurring, respectively. Additionally, the models estimated overall occupancy, or the probability that bats were present at a site regardless of state (*ψ*^*1*^), the conditional probability that state 2 (high activity or feeding) actually occurred given bat presence (*ψ*^*2*^; the parameter of primary interest), and the probability of correctly identifying state 2 versus state 1 given the detection of bat presence (*δ*).

A key assumption in occupancy modeling is that the occupancy state (e.g., not detected, present at low abundance, present at high abundance) remains constant for the duration of the sampling period [7]. When sample plot areas are smaller than an individual’s home range and movement leads to temporary absence, occupancy estimates should instead be interpreted as the ‘proportion of area used’ (or for multi-state model parameterizations, ‘probability of site use’), rather than as the proportion of area occupied [46, 47]. MacKenzie et al. [14] caution that the relaxation of the assumption of site closure may introduce bias. However, they do so only in the context of directional changes in the occupancy state over a survey period (e.g., seasonal trend in the breeding calls of frogs). We do not believe that Hawaiian hoary bats are likely to exhibit seasonal trends in foraging rates over the short time period (4 nights) at which it was measured at each site. MacKenzie et al. [14] also note “[w]hen such changes occur at random … the interpretation of the state-specific occupancy-related probabilities is the probability that that state is the highest reached during the season (i.e., the state of a unit may not always be 2, but 2 is the highest state reached at some point during the surveying)”. We interpret our results along these lines such that parameter *ψ*^*2*^ represents the mean probability that state 2 actually occurred at sample sites at some point during the survey, and represents the extent to which the survey area as a whole can support high levels of bat activity and feeding; i.e., “focal” habitat use. Furthermore, it is important to note that relaxation of the site closure assumption also applies to the interpretation of parameter *ψ*^*1*^. That is, the parameter indicates the probability, regardless of actual state, that one or more bats were present at some point at a site during a survey period. For the totality of sites, it represents the overall prevalence of the organism in the surveyed area inclusive of “non-focal” habitat use (e.g., transit between sites).

Low and high categories of activity were distinguished based on the median detection rates from acoustic and video data obtained from a previous 1-year bat study in the same region [38]. To distinguish high from low activity, the thresholds for acoustic and video data was set at a nightly count of 1.0 and 5.0 per site, respectively. Counts greater than those values were designated as “2” in the detection history matrix used in the multi-state analyses, with “1” indicating observations of only low activity or no feeding, and “0” representing no observations of bats. The choice of the median as a threshold was arbitrary but served in our study as an example of how it may be used to identify higher-use areas; different threshold values can be obtained from pilot studies or other knowledge of a species’ biology.

Multi-state model parameters can be fit with and without covariates. The basic occupancy model that included only their intercepts without covariates served as a null model against which we compared model types and covariate-fitted models. The null model specified separate parameters for the detection probabilities of state 1 and state 2, and is referred to herein as “null” with the notation *ψ*^*1*^(·)*ψ*^*2*^(·)*p*^*1*^(·)*p*^*2*^(·)*δ*(·). To restrict the number of candidate models and likelihood of obtaining spurious results given a limited sample size, and given our study’s focus on the relationship between insect prey and bat presence, we constrained our models to include only insect sample covariates that might affect overall prevalence (*ψ*^*1*^) and the occurrence of high activity or feeding (*ψ*^*2*^). We did not examine the use of site-specific covariate data in multi-state models because the survey area was limited to 25 sq km of relatively homogenous land-cover and physiognomic attributes that are readily traversed by resident bats (e.g., Hawaiian hoary bats tracked by radio telemetry had long-axis home range movements up to 18 km [mean = 3.4 km ± 0.8 SE]; [48]). Post-hoc trials with “single-state” occupancy models also did not demonstrate any support for the inclusion of sample-specific covariates (which can change across sampling nights) comprising mean wind speed, wind variability, total precipitation, proportion of night with rain, and night-time sky illumination. The trials and consistent mild weather during the sampling period justified the exclusion of these variables from multi-state models (although such covariates can be readily included where sites attributes differ spatially and sampling conditions vary over a survey period). Therefore, we estimated *p*^1^ and *p*^2^ as constant over the 4 consecutive nights of sampling at each site. Similarly, survey night was not deemed likely to affect the probability of correctly identifying state 2 so *δ* was also treated as a constant over all samples.

Mean nightly Coleoptera and Lepidoptera biomass were each considered as site covariates that could independently affect bat presence (if bats preferred one food type over the other) and are referred to in models as “beetle” and “moth”, respectively. We also included the combined Coleoptera and Lepidoptera biomass as an additional site covariate reflective of overall site productivity (“insect”). For models which used these covariates only for the parameter *ψ*^*1*^, the hypothesis was that prey biomass affects overall bat prevalence but not the probability of high bat activity or feeding behavior. Conversely, where parameter *ψ*^*1*^ was treated as a constant and parameter *ψ*^*2*^ included covariates, the interpretation was that prey biomass determines high bat activity or feeding behavior but was not related to overall prevalence. Finally, where *ψ*^*1*^ included the covariate “insect” and parameter *ψ*^*2*^ included either “beetle” or “moth”, the models assumed that prevalence was determined by site productivity, but the likelihood of high bat activity or feeding at a site depended on bat preference for either beetles or moths.

We used the program PRESENCE version 12.7 [49] to obtain maximum likelihood estimates for model parameters and rankings. The number of sites (n = 20) was used as the effective sample size to calculate the small-sample-corrected Akaike's Information Criterion (AIC_c_) and relative model weights reflect evidence in favor of the respective models being the most appropriate among the members of the model set [50]. For comparability, we initially present null model (intercept-only) parameter estimates for all four model types (acoustic–activity, acoustic–feeding, video–activity, video–feeding), and subsequently examine covariate-fitted models that rank better than the null for inference of bat-prey relationships. Parameter estimates are provided with their unconditional standard errors. Bat detection histories for each of the four model types are tabulated in S2 Table, and site samples of prey biomass are presented in S1 Table and S1 Fig. Acoustic and video detections of bats are shown in S2 and S3 Figs.

## Results

Acoustic bat detections were relatively infrequent and averaged 0.8 per night/site (range = 0–5; median = 0.0), whereas video sampling resulted in an average of 5.5 detections per night/site (range = 0–35; median = 7.2). Acoustic detections were recorded at 13 of the 20 sites, yielding a naïve occupancy probability of ${\tilde{\psi }}^{1}$ = 0.65 (where “naïve” means not accounting for imperfect detection). High activity and feeding were detected acoustically at least once at 7 and 6 sites known to be occupied, respectively, yielding naïve estimates of ${\tilde{\psi }}^{2}$ = 0.35 and 0.30 for the prevalence of these events. In comparison, bats were detected by video at all sites during the survey; that is, ${\tilde{\psi }}^{1}$ = 1.0. High activity and feeding were also detected by video at least once at 15 and 16 sites known to be occupied, respectively, yielding naïve estimates of ${\tilde{\psi }}^{2}$ = 0.75 and 0.80 for the prevalence of these events.

The probability that a site was occupied, ${\hat{\psi }}^{1}$, was estimated as 1 by all null models (Table 1), a result substantially higher than the naïve estimates but in agreement with the scenario that Hawaiian hoary bats occur across all sites in the study area regardless of the sparsity of detections related to activity or feeding. Estimates of ${\hat{p}}^{1}$, the probability of detecting bats in state 1 (low activity or no observed feeding), for each of the two survey methods was similar in terms of the type of bat observation (activity versus feeding) but differed between sampling methods, with the video-based method demonstrating much higher parameter values. Of greater interest, video samples yielded higher estimates and greater precision for both ${\hat{p}}^{2}$ and ${\hat{\psi }}^{2}$ compared to acoustic methods. For example, the probability of detecting high bat activity by video was almost twice that obtained by acoustic sampling: ${\hat{p}}^{2}$ = 0.96 (SE = 0.028) versus 0.52 (SE = 0.116). Likewise, the video-derived estimate of the prevalence of feeding, ${\hat{\psi }}^{2}$, was 0.89 (SE = 0.108) compared to 0.48 (SE = 0.205) for acoustic methods.

**Table 1:** Null model parameter estimates for each of four model types.

	Acoustic–activity		Acoustic–feeding		Video–activity		Video–feeding	
Parameter	Mean	SE	Mean	SE	Mean	SE	Mean	SE
${\hat{\psi }}^{1}$	1.00	†	1.00	†	1.00	†	1.00	†
${\hat{\psi }}^{2}$	0.48	0.161	0.48	0.205	0.78	0.103	0.89	0.108
${\hat{p}}^{1}$	0.19	0.076	0.17	0.078	0.83	0.101	0.86	0.156
${\hat{p}}^{2}$	0.52	0.116	0.70	0.273	0.98	0.021	0.96	0.028
$\hat{\delta }$	0.75	0.131	0.32	0.118	0.74	0.101	0.49	0.073

† Standard error (SE) cannot be calculated for parameters estimated at boundary of parameter space.

*ψ*^*1*^—probability that bats were present at a site regardless of state (high activity or feeding)

*ψ*^*2*^—conditional probability that state 2 actually occurred given bat presence

*p*^*1*^—probability of observing the species in state 1 given its true state was 1

*p*^*2*^—probability of observing the species in state 2 given its true state was 2

*δ* —probability of correctly identifying state 2 versus state 1 given the detection of bat presence.

The probability of correctly classifying high activity given bat presence, $\hat{\delta }$, was one and a half to twice that of identifying feeding events, irrespective of sampling method. For example, $\hat{\delta }$ = 0.74 (SE = 0.101) for video samples of bat activity, and indicated that there was a 74% chance of a nightly sample visually recording >5 separate detections. A similar result was evident from acoustic samples of bat activity. Finally, both acoustic and video-based samples of feeding activity exhibited fairly low values ($\hat{\delta }$ = 0.32 and 0.49, respectively), suggesting that the probability of correctly identifying feeding from either method may be difficult and liable to underestimate true *ψ*^*2*^. Nonetheless, estimates of *ψ*^*2*^ for all null models were higher than the naïve estimates, a result consistent with the expectation that occupancy models adjust upward appraisals of occurrence when detection probabilities are <1.

Model selection statistics indicated that null model weights made up the plurality or majority of the support for three of the four model types (S3 Table). The exception was for the acoustic–activity set in which the three top models ranked better than the null model and together comprised 93% of total weight. The models *ψ*^*1*^(·)*ψ*^*2*^(beetle)*p*^1^(·)*p*^2^(·)*δ*(·) and *ψ*^*1*^(·)*ψ*^*2*^(insect)*p*^1^(·)*p*^2^(·)*δ*(·) each demonstrated that the probability of high acoustic activity occurring at a site was positively related to beetle and insect biomass, respectively (Table 2). The model *ψ*^*1*^(insect)*ψ*^*2*^(beetle)*p*^1^(·)*p*^2^(·)*δ*(·) showed that overall bat prevalence was dependent on insect abundance, whereas the probability of high acoustic activity was related to beetle biomass. In keeping with these results, site-specific estimates of ${\hat{\psi }}^{2}$from the top model averaged only 0.39 for the study area as a whole and revealed that high acoustic activity was not widespread, yet the estimates were fairly well correlated with Coleoptera biomass samples (r = 0.78) and suggest that high vocalization rates may be linked to available prey (S4 Fig). Non-native coprophagous dung beetles (Scarabaeidae, *Onthophagus* spp.) were the most common group of Coleoptera encountered, with high proportions of the nocturnally volant *O*. *sagittarius* prevalent in the light trap samples.

**Table 2:** Parameter estimates for top ranked models of the acoustic–activity set. Estimates for *ψ*^*2*^ (the probability that state 2 –high activity or feeding–actually occurred given bat presence) were obtained by averaging site-specific predicted values and their standard errors. Parameter definitions are provided in the footnote to Table 1.

	*ψ*^*1*^(·)*ψ*^*2*^(beetle)*p*^1^(·)*p*^2^(·)*δ*(·)		*ψ*^*1*^(·)*ψ*^*2*^(insect)*p*^1^(·)*p*^2^(·)*δ*(·)		*ψ*^*1*^(insect)*ψ*^*2*^(beetle)*p*^1^(·)*p*^2^(·)*δ*(·)	
Parameter	Mean	SE	Mean	SE	Mean	SE
${\hat{\psi }}^{1}$	1.00	†	1.00	†	1.00	†
${\hat{\psi }}^{2}$	0.39	0.123	0.39	0.126	0.40	0.130
${\hat{p}}^{1}$	0.20	0.069	0.20	0.068	0.22	0.078
${\hat{p}}^{2}$	0.56	0.111	0.56	0.109	0.56	0.113
$\hat{\delta }$	0.79	0.107	0.79	0.107	0.79	0.107

† Standard error (SE) cannot be calculated for parameters estimated at boundary of parameter space.

## Discussion

Our study is the first application of multi-state occupancy analysis of bat activity and behavior for the purpose of evaluating habitat use and quality, an assessment that can be particularly difficult to quantify for bat species. The use of this type of model in bat ecology is novel and our study may serve to inform its application to other such investigations. For example, our study demonstrated that the probability of detecting high bat activity and feeding by video was high or close to 1.0 in null models, which may argue for the use of analyses that do not require correction for imperfect detection for this type of data. Alternatively, given the interpretation of habitat use based on cumulative samples of occurrence, it also indicates that fewer video samples at sites expected to have a high frequency of detections of interest may suffice to achieve a desired level of precision in occupancy model parameters. The use of both acoustic and video sampling methods also provided a useful comparison from which to gauge in subsequent studies the relative effort necessary to balance the number of sites and repeat samples in light of the preferred sampling method [51].

Small sample sizes are common to ecological studies and conservation projects, which typically have limited resources and focus on rare species. Such studies may benefit from a larger number of samples than that available in this study to reduce the risk of over-fitting models and to accommodate covariates in addition to prey biomass. We also caution that models are expected to perform better where estimated parameters do not attain a maximum value of 1.0 and preclude estimation of standard error (i.e., “boundary effect”). This effect was evident in estimates of *ψ*^*1*^ which indicated that the study area was “saturated” and the actual prevalence of bats was widespread (more so than anticipated during the study design). However, in our case this was not relevant given that focal habitat use as estimated by *ψ*^*2*^ is the parameter of primary interest and detection of high activity and feeding was not ubiquitous.

It is important to note that multi-state occupancy models do not include a separate parameter to estimate availability, that is, the occurrence of individuals exposed to sampling and available for detection. Despite this, the non-identifiability of detection and availability components is not an issue where availability is relatively constant. Although the sample volume of both acoustic and video devices are considerably smaller than the total area used nightly by individual bats, the overlap of these areas and sample sites is not likely to change over short time periods. We believe this condition is met, in part, by sampling at a “fine-grained” temporal resolution over periods of short duration (in our study, nightly samples over 4 nights). MacKenzie et al. [52] demonstrated a similar approach by sampling amphibian activity during a fixed period during which species were active and known to be consistently available for detection. Moreover, a telemetry study of Hawaiian hoary bats [48] demonstrated high foraging site fidelity in which individuals make repeated nightly use of areas within periods of at least several weeks. This, and the energetic need for bats to forage nightly (particularly during the breeding season) and general ubiquity (estimated overall prevalence was equal to 1), supports our assumption that their availability for detection is fairly invariant over short periods.

Efford and Dawson [53] distinguish asymptotic occupancy, the accumulated area used over time, from that of instantaneous occupancy, the proportion of sites occupied at a point in time. The distinction between use and occupancy is important in that it highlights the need to consider both the duration of sampling and the spatial extent of sampling if comparisons are to be made among surveys. For our study, multi-state models produced useful metrics describing the prevalence of high bat activity and feeding, results which can be used to track trends in habitat use and quality within this particular study area. Similar surveys can be applied elsewhere but the spatial and temporal grain of sampling (i.e., plot size relative to study area), survey duration and number of samples over time should be consistent if comparisons among areas or over time is the objective.

In terms of habitat use and inferences regarding habitat quality, models demonstrated that elevated levels of acoustic activity (i.e. number of calls detected nightly) by Hawaiian hoary bats were related primarily to beetle biomass at this particular place and time. Insect biomass as a covariate may also have ranked highly in acoustic–activity models because beetles comprised about three-quarters of the combined Coleoptera-Lepidotera biomass (S4 Table). Models with moth biomass as a covariate did not perform better than null models in any of the four model types, a result that might have been due to moths comprising a relatively minor amount of the insects sampled. It is possible that a relationship between Hawaiian hoary bat foraging activity and moth abundance might be more evident where and when moth availability is higher. For example, moths were the most abundant insect order and dominated the diet of Hawaiian hoary bats in open habitat, but were consumed less in cluttered habitat where they were generally smaller than the minimum prey size noted in a study on Kaua‘i Island [35]. In addition, moth and beetle prevalence in the diet of hoary bats in North America appears to be seasonally variable, indicating some degree of opportunistic feeding [54–55].

Bat detection rates were higher for video than for acoustic sampling methods, but neither the video–activity nor video–feeding model sets resulted in models with moth, beetle or insect covariates that ranked better than the null. Although null model mean estimates *p*^2^ and *ψ*^*2*^ were considerably higher and precision was greater for video-based methods, measures of elevated acoustic activity appeared to be more closely associated with prey abundance. This may reflect the possibility that the higher rates of bat detections by video include commuting individuals not engaged in foraging situations. Recent observations of hoary bats not detectably vocalizing while in flight [5,6] suggest that the species may not be entirely reliant on echolocation for nocturnal navigation. Consequently, when vocalization is detected may be a more reliable indicator of prey targeting and active foraging than the number of flight passes detected visually.

Feeding activity as identified by terminal-phase calls (“feeding buzzes”) were not demonstrably related to beetle or moth biomass, but this result may reflect the relative sparsity of these particular detections (only once at each of 6 sites over the 61 nights with acoustic samples) rather than the actual absence of such relationship. Terminal-phase calls are unambiguous indicators of prey targeting and likely feeding, but are typically emitted at a much lower intensity than search or approach phase calls so as to temper gain and prevent self-deafening from echoes as a bat closes in on a target [56–58]. As such, terminal-phase calls are more difficult to detect in the field and using these calls as a measure of feeding may under-represent these events compared to tallies obtained from the acoustics of bats simply searching for prey. Notably, of the four model types, the acoustic–feeding model exhibited the highest uncertainty in correctly identifying feeding activity ($\hat{\delta }$ = 0.32; Table 1).

As with the detection of terminal-phase calls, identifying flight trajectories that included a rapid loop or roll from video recordings may undercount the actual prevalence of feeding events because Hawaiian hoary bats may be able to catch prey without such manoeuvers. Therefore, although observed detection probability was quite high (${\hat{p}}^{2}$ = 0.96; Table 1), the state assignment was characterized by uncertainty ($\hat{\delta }$ = 0.49) and likely hindered modeling the relationships between the prevalence of this behavior and beetle and moth biomass.

The strength of bat activity and prey biomass associations can be expected to change across habitats and seasons in response to shifts in the composition and abundance of available insects. The apparent absence of bat activity and prey biomass associations for three of the four model types may be partly due to prey not actually being a limiting resource in the summer months when insect abundance is generally highest. Even reproductive bats with higher energetic demands likely can achieve a positive energy balance after only a few hours of foraging [59]. Consequently, causal factors in the spatial patterns of bat abundance, food resources, and insect predation may only be evident during seasons or periods of less favorable environmental conditions and more restricted resource availability.

Multi-state modeling has the potential to be useful in studies where investigators can obtain information at occupied sites about species status (e.g., encounter rates, behavior, etc.). Information about bat activity and the availability of foraging resources can be acquired for many species and settings, and may often be obtained non-invasively and with less effort than comparable sampling involving bat capture and handling. Assuming that sites with higher abundances, activity levels or specific behaviors (e.g. feeding or breeding) are indicative of higher habitat quality or availability, the opportunity exists to use these models to explore relationships between species status and focal resources. Multi-state occupancy modeling also can be applied to multi-season models to assess trends in specific habitat use that might not be apparent solely from short-term assessments of species presence [60]. For example, overall prevalence (as represented by *ψ*^*1*^) of a widely ranging species may be relatively constant over time, yet the proportion of sites at which focal species activity or behavior is tracked could change in directions important to conservation management (e.g., habitat restoration, resource extraction impacts, etc.). Such models can allow inference about transition probabilities for both species occupancy state and habitat state, and the dependence of species status on habitat condition.

In summary, multi-state occupancy modeling can establish quantitative relationships in settings where sampling is difficult and animal crypsis results in imperfect detection. Although applied to coarse categories of bat activity and behavior, the resulting models are robust and explicitly incorporate uncertainty. Krebs [61] stated that "Ecology is the scientific study of the interactions that determine the distribution and abundance of organisms. We are interested in where organisms are found, how many occur there, and why". By linking animal activity, occurrence and habitat, multi-state modeling can use information of the *how many* (or how much) to effectively describe the *where* and infer the *why*.

## Supporting information

S1 Table Mean weight by site for samples used as covariates “beetle” (Coleoptera), “moth” (Lepidoptera) and “insect” (combined Coleoptera and Lepidoptera weights) in the multi-state occupancy models. (DOCX)

S2 Table Bat detection histories by site and nightly sample for the four multi-state occupancy model types: Acoustic–activity, Acoustic–feeding, Video–activity, and Video–feeding. (DOCX)

S3 Table Model selection statistics for 9 multi-state occupancy models fit to each of four model types: Acoustic–activity, Acoustic–feeding, Video–activity, and Video–feeding. (DOCX)

S4 Table Total weight (panel a) and count (panel b) and associated proportions (prop) of arachnid and insect samples recorded over 4 nights at each of 20 sites from 10 July to 10 August 2017 in the northern Ko‘olau Mountains of O‘ahu.
(DOCX)

S1 Fig Coleoptera and Lepidoptera biomass (mean nightly dry weight; grams) samples by site. (DOCX)

S2 Fig Acoustic detections by site of Hawaiian hoary bats (*Lasiurus cinereus semotus*) and the subset identified as comprising feeding behavior (terminal-phase calls). (DOCX)

S3 Fig Videographic detections by site of Hawaiian hoary bats (*Lasiurus cinereus semotus*) and the subset identified as comprising feeding behavior. (DOCX)

S4 Fig Sampled Coleoptera biomass and the predicted probability of high acoustic activity by Hawaiian hoary bats (*Lasiurus cinereus semotus*) given that the site is occupied (occupancy state 2). (DOCX)
