# Supplementary material for: Multi-state occupancy models of foraging habitat use by the Hawaiian hoary bat (Lasiurus cinereus semotus)
Source: PLoS One. 2018 Oct 31;13(10):e0205150. doi: 10.1371/journal.pone.0205150 (PMC6209161; doi:10.1371/journal.pone.0205150)
Supplement: S1 Table — (DOCX) [file pone.0205150.s001.docx]

S1 Table. Mean weight (grams) by site for samples used as covariates “beetle” (Coleoptera), “moth” (Lepidoptera) and “insect” (combined Coleoptera and Lepidoptera weights) in the multi-state occupancy models. Insect samples were recorded over 4 nights each at 20 sites from 10 July to 10 August 2017 in the northern Ko‘olau Mountains of O‘ahu. Site locations are given as Universal Transverse Mercator coordinates (Northing, Easting) using a Zone 4 North projection and NAD83 datum.

| Site | Northing | Easting | Beetle | Moth | Insect |
| --- | --- | --- | --- | --- | --- |
| 1 | 2389631 | 600965 | 0.1429 | 0.3283 | 0.4712 |
| 2 | 2391459 | 599033 | 0.1283 | 0.2440 | 0.3723 |
| 3 | 2388746 | 598456 | 0.8729 | 0.2054 | 1.0782 |
| 4 | 2389008 | 600482 | 0.8165 | 0.3430 | 1.1595 |
| 5 | 2390991 | 599855 | 0.1751 | 0.1486 | 0.3237 |
| 6 | 2391882 | 598327 | 0.0118 | 0.0394 | 0.0512 |
| 7 | 2387956 | 600493 | 1.7214 | 0.6774 | 2.3988 |
| 8 | 2390093 | 597749 | 1.1239 | 0.3636 | 1.4875 |
| 9 | 2390268 | 600565 | 0.2587 | 0.4641 | 0.7228 |
| 10 | 2390208 | 598606 | 3.8318 | 0.2447 | 4.0765 |
| 11 | 2387801 | 599864 | 0.6332 | 0.2471 | 0.8803 |
| 12 | 2388064 | 598473 | 0.8419 | 0.1722 | 1.0142 |
| 13 | 2390018 | 599383 | 0.0721 | 0.1737 | 0.2458 |
| 14 | 2391030 | 597498 | 0.2267 | 0.0745 | 0.3012 |
| 15 | 2388375 | 601308 | 0.2175 | 0.2724 | 0.4899 |
| 16 | 2389286 | 598285 | 2.0879 | 0.0882 | 2.1761 |
| 17 | 2389818 | 600084 | 0.3217 | 0.1910 | 0.5127 |
| 18 | 2390567 | 597888 | 0.3344 | 0.1160 | 0.4504 |
| 19 | 2388762 | 601754 | 0.0528 | 0.3196 | 0.3724 |
| 20 | 2388975 | 598948 | 0.6902 | 0.2690 | 0.9592 |
