# Supplementary material for: Multi-state occupancy models of foraging habitat use by the Hawaiian hoary bat (Lasiurus cinereus semotus)
Source: PLoS One. 2018 Oct 31;13(10):e0205150. doi: 10.1371/journal.pone.0205150 (PMC6209161; doi:10.1371/journal.pone.0205150)
Supplement: S2 Table — (DOCX) [file pone.0205150.s002.docx]

S2 Table. Bat detection histories by site and nightly sample for the four multi-state occupancy model types: Acoustic–activity, Acoustic–feeding, Video–activity, and Video–feeding. Notation: "0" = no detection, "1" = detection with no evidence of high activity or feeding, "2" = detection with evidence of high activity or feeding, "-" = missing data. Bat detection histories were recorded over 4 nights each at 20 sites from 10 July to 10 August 2017 in the northern Ko‘olau Mountains of O‘ahu. Site locations are presented in S1 Table.

| Site |  | Acoustic–activity | | | |  | Acoustic–feeding | | | |  | Video–activity | | | |  | Video–feeding | | | |
| --- | --- | --- | --- | --- | --- | --- | --- | --- | --- | --- | --- | --- | --- | --- | --- | --- | --- | --- | --- | --- |
| 1 |  | 0 | 0 | - | - |  | 0 | 0 | - | - |  | 2 | 2 | 1 | 1 |  | 2 | 2 | 2 | 1 |
| 2 |  | - | - | - | - |  | - | - | - | - |  | 2 | 2 | 1 | 2 |  | 2 | 2 | 2 | 2 |
| 3 |  | 0 | 2 | - | - |  | 0 | 1 | - | - |  | 2 | 2 | 2 | 2 |  | 2 | 2 | 2 | 1 |
| 4 |  | 0 | 0 | - | - |  | 0 | 0 | - | - |  | 2 | 2 | 2 | 2 |  | 2 | 2 | 2 | 2 |
| 5 |  | 0 | 0 | 0 | 1 |  | 0 | 0 | 0 | 1 |  | 2 | 2 | 1 | 2 |  | 1 | 2 | 2 | 1 |
| 6 |  | 0 | 0 | 0 | 0 |  | 0 | 0 | 0 | 0 |  | 2 | 2 | 2 | 2 |  | 2 | 1 | 1 | 2 |
| 7 |  | 1 | 2 | 0 | 0 |  | 1 | 1 | 0 | 0 |  | 2 | 2 | 1 | - |  | 2 | 2 | 1 | - |
| 8 |  | 0 | 2 | 0 | - |  | 0 | 1 | 0 | - |  | 2 | 2 | 1 | - |  | 2 | 2 | 2 | - |
| 9 |  | 1 | 0 | 0 | 0 |  | 1 | 0 | 0 | 0 |  | 1 | 1 | 1 | 1 |  | 1 | 1 | 1 | 1 |
| 10 |  | 1 | 2 | 2 | 2 |  | 1 | 1 | 1 | 2 |  | 2 | 2 | 1 | 1 |  | 1 | 1 | 1 | 2 |
| 11 |  | 0 | 1 | 0 | 0 |  | 0 | 1 | 0 | 0 |  | 1 | 1 | 1 | 2 |  | 1 | 1 | 1 | 1 |
| 12 |  | 0 | 0 | 0 | 0 |  | 0 | 0 | 0 | 0 |  | 2 | 1 | 1 | 2 |  | 1 | 1 | 2 | 1 |
| 13 |  | 0 | 1 | 0 | - |  | 0 | 2 | 0 | - |  | 2 | 1 | 1 | 0 |  | 2 | 1 | 2 | 0 |
| 14 |  | 1 | 1 | 1 | - |  | 1 | 1 | 2 | - |  | 1 | 1 | 0 | 1 |  | 1 | 2 | 0 | 1 |
| 15 |  | 1 | 0 | 0 | 0 |  | 2 | 0 | 0 | 0 |  | 1 | 0 | - | - |  | 1 | 0 | - | - |
| 16 |  | 2 | 1 | 2 | 2 |  | 1 | 2 | 1 | 1 |  | 1 | 1 | 2 | 1 |  | 1 | 1 | 2 | 1 |
| 17 |  | 1 | 0 | 2 | 2 |  | 1 | 0 | 1 | 2 |  | 2 | 2 | 1 | 1 |  | 2 | 1 | 1 | 1 |
| 18 |  | 0 | 0 | - | - |  | 0 | 0 | - | - |  | 1 | 1 | 1 | 0 |  | 1 | 1 | 1 | 0 |
| 19 |  | 0 | - | - | - |  | 0 | - | - | - |  | 1 | 1 | 1 | 2 |  | 1 | 2 | 1 | 1 |
| 20 |  | 0 | 0 | 2 | 0 |  | 0 | 0 | 1 | 0 |  | 1 | 1 | 1 | 1 |  | 2 | 1 | 1 | 1 |
