# Supplementary material for: Multi-state occupancy models of foraging habitat use by the Hawaiian hoary bat (Lasiurus cinereus semotus)
Source: PLoS One. 2018 Oct 31;13(10):e0205150. doi: 10.1371/journal.pone.0205150 (PMC6209161; doi:10.1371/journal.pone.0205150)
Supplement: S3 Table — (DOCX) [file pone.0205150.s003.docx]

S3 Table. Model selection statistics for 9 multi-state occupancy models fit to each of four model types: Acoustic–activity, Acoustic–feeding, Video–activity, and Video–feeding.

| Acoustic – activity | | | | |
| --- | --- | --- | --- | --- |
| Model | Parameters | -2*L* | ΔAICc | Weight |
| psi(.) R(beetle) p1(.) p2(.) delta(.) | 6 | 96.23 | 0.00 | 0.45 |
| psi(.) R(insect) p1(.) p2(.) delta(.) | 6 | 96.56 | 0.33 | 0.38 |
| psi(insect) R(beetle) p1(.) p2(.) delta(.) | 7 | 94.29 | 2.93 | 0.10 |
| psi(.) R(.) p1(.) p2(.) delta(.) NULL | 5 | 106.55 | 6.14 | 0.02 |
| psi(beetle) R(.) p1(.) p2(.) delta(.) | 6 | 102.65 | 6.42 | 0.02 |
| psi(insect) R(.) p1(.) p2(.) delta(.) | 6 | 103.60 | 7.37 | 0.01 |
| psi(moth) R(.) p1(.) p2(.) delta(.) | 6 | 103.60 | 7.37 | 0.01 |
| psi(.) R(moth) p1(.) p2(.) delta(.) | 6 | 105.61 | 9.38 | <0.01 |
| psi(insect) R(moth) p1(.) p2(.) delta(.) | 7 | 103.09 | 11.73 | <0.01 |

| Acoustic – feeding | | | | |
| --- | --- | --- | --- | --- |
| Model | Parameters | -2*L* | ΔAICc | Weight |
| psi(.) R(.) p1(.) p2(.) delta(.) NULL | 5 | 107.05 | 0.00 | 0.23 |
| psi(beetle) R(.) p1(.) p2(.) delta(.) | 6 | 103.11 | 0.24 | 0.21 |
| psi(insect) R(.) p1(.) p2(.) delta(.) | 6 | 104.16 | 1.29 | 0.12 |
| psi(moth) R(.) p1(.) p2(.) delta(.) | 6 | 104.16 | 1.29 | 0.12 |
| psi(.) R(moth) p1(.) p2(.) delta(.) | 6 | 104.33 | 1.46 | 0.11 |
| psi(.) R(beetle) p1(.) p2(.) delta(.) | 6 | 105.33 | 2.46 | 0.07 |
| psi(insect) R(moth) p1(.) p2(.) delta(.) | 7 | 100.71 | 2.71 | 0.06 |
| psi(.) R(insect) p1(.) p2(.) delta(.) | 6 | 105.61 | 2.74 | 0.06 |
| psi(insect) R(beetle) p1(.) p2(.) delta(.) | 7 | 103.02 | 5.02 | 0.02 |

| Video – activity | | | | |
| --- | --- | --- | --- | --- |
| Model | Parameters | -2*L* | ΔAICc | Weight |
| psi(.) R(.) p1(.) p2(.) delta(.) NULL | 5 | 124.53 | 0.00 | 0.48 |
| psi(.)R(beetle) p1(.) p2(.) delta(.) | 6 | 122.89 | 2.54 | 0.13 |
| psi(.) R(insect) p1(.) p2(.) delta(.) | 6 | 122.97 | 2.62 | 0.13 |
| psi(.) R(moth) p1(.) p2(.) delta(.) | 6 | 124.41 | 4.06 | 0.06 |
| psi(beetle) R(.) p1(.) p2(.) delta(.) | 6 | 124.53 | 4.18 | 0.06 |
| psi(moth) R(.) p1(.) p2(.) delta(.) | 6 | 124.53 | 4.18 | 0.06 |
| psi(insect) R(.) p1(.) p2(.) delta(.) | 6 | 124.53 | 4.18 | 0.06 |
| psi(insect) R(beetle) p1(.) p2(.) delta(.) | 7 | 122.89 | 7.41 | 0.01 |
| psi(insect) R(moth) p1(.) p2(.) delta(.) | 7 | 124.41 | 8.93 | 0.01 |

| Video – feeding | | | | |
| --- | --- | --- | --- | --- |
| Model | Parameters | -2*L* | ΔAICc | Weight |
| psi(.) R(.) p1(.) p2(.) delta(.) NULL | 5 | 129.15 | 0.00 | 0.54 |
| psi(.) R(beetle) p1(.) p2(.) delta(.) | 6 | 128.45 | 3.48 | 0.09 |
| psi(.) R(insect) p1(.) p2(.) delta(.) | 6 | 128.63 | 3.66 | 0.09 |
| psi(.) R(moth) p1(.) p2(.) delta(.) | 6 | 129.10 | 4.13 | 0.07 |
| psi(beetle) R(.) p1(.) p2(.) delta(.) | 6 | 129.15 | 4.18 | 0.07 |
| psi(moth) R(.) p1(.) p2(.) delta(.) | 6 | 129.15 | 4.18 | 0.07 |
| psi(insect) R(.) p1(.) p2(.) delta(.) | 6 | 129.15 | 4.18 | 0.07 |
| psi(insect) R(beetle) p1(.) p2(.) delta(.) | 7 | 128.45 | 8.35 | 0.01 |
| psi(insect) R(moth) p1(.) p2(.) delta(.) | 7 | 129.10 | 9.00 | 0.01 |
