# Supplementary material for: Multi-state occupancy models of foraging habitat use by the Hawaiian hoary bat (Lasiurus cinereus semotus)
Source: PLoS One. 2018 Oct 31;13(10):e0205150. doi: 10.1371/journal.pone.0205150 (PMC6209161; doi:10.1371/journal.pone.0205150)
Supplement: S4 Table — Total weight (panel a) and count (panel b) and associated proportions (prop) of arachnid and insect samples recorded over 4 nights at each of 20 sites from 10 July to 10 August 2017 in the northern Ko‘olau Mountains of O‘ahu. (DOCX) [file pone.0205150.s004.docx]

S4 Table. Total weight (panel a) and count (panel b) and associated proportions (prop) of arachnid and insect samples recorded over 4 nights at each 20 sites from 10 July to 10 August 2017 in the northern Ko‘olau Mountains of O‘ahu.

1. Dry weight (grams)

|  | Medium (5-10 mm) | |  | Large (>10-20 mm) | |  | Extra-large (> 20 mm) | |
| --- | --- | --- | --- | --- | --- | --- | --- | --- |
| Order | weight | prop |  | weight | prop |  | weight | prop |
| Araneae | 0.073 | 0 |  | 0 | 0 |  | 0.717 | 0.04 |
| Blattodea | 0.102 | 0 |  | 0.236 | 0.01 |  | 0.441 | 0.02 |
| Coleoptera | 30.430 | 0.69 |  | 22.200 | 0.59 |  | 0.889 | 0.05 |
| Dermaptera | 0.130 | 0 |  | 0.210 | 0.01 |  | 0.180 | 0.01 |
| Diptera | 0.651 | 0.01 |  | 0.155 | 0 |  | 0 | 0 |
| Hemiptera | 0.613 | 0.01 |  | 0.601 | 0.02 |  | 0.023 | 0 |
| Homoptera | 1.619 | 0.04 |  | 0 | 0 |  | 0 | 0 |
| Hymenoptera | 0.705 | 0.02 |  | 0.512 | 0.01 |  | 0 | 0 |
| Isoptera | 1.766 | 0.04 |  | 0.130 | 0 |  | 0 | 0 |
| Lepidoptera | 7.418 | 0.17 |  | 10.921 | 0.29 |  | 11.706 | 0.65 |
| Mantodea | 0.031 | 0 |  | 0.058 | 0 |  | 0.532 | 0.03 |
| Orthoptera | 0.270 | 0.01 |  | 2.705 | 0.07 |  | 3.609 | 0.20 |
| Zoraptera | 0.022 | 0 |  | 0 | 0 |  | 0 | 0 |
| sum | 43.826 |  |  | 37.722 |  |  | 18.096 |  |

1. Count

|  | Medium (5-10 mm) | |  | Large (>10-20 mm) | |  | Extra-large (> 20 mm) | |
| --- | --- | --- | --- | --- | --- | --- | --- | --- |
| Order | count | prop |  | count | prop |  | count | prop |
| Araneae | 5 | 0 |  | 0 | 0 |  | 5 | 0.05 |
| Blattodea | 5 | 0 |  | 11 | 0.01 |  | 9 | 0.08 |
| Coleoptera | 1,443 | 0.24 |  | 552 | 0.49 |  | 8 | 0.07 |
| Dermaptera | 10 | 0 |  | 12 | 0.01 |  | 3 | 0.03 |
| Diptera | 295 | 0.05 |  | 7 | 0.01 |  | 0 | 0 |
| Hemiptera | 161 | 0.03 |  | 12 | 0.01 |  | 1 | 0.01 |
| Homoptera | 490 | 0.08 |  | 2 | 0 |  | 0 | 0 |
| Hymenoptera | 524 | 0.09 |  | 17 | 0.02 |  | 0 | 0 |
| Isoptera | 299 | 0.05 |  | 8 | 0.01 |  | 0 | 0 |
| Lepidoptera | 2,692 | 0.45 |  | 414 | 0.37 |  | 52 | 0.48 |
| Mantodea | 1 | 0 |  | 1 | 0 |  | 5 | 0.05 |
| Orthoptera | 67 | 0.01 |  | 85 | 0.08 |  | 25 | 0.23 |
| Zoraptera | 2 | 0 |  | 0 | 0 |  | 0 | 0 |
| sum | 5,994 |  |  | 1,121 |  |  | 108 |  |
