# Supplementary figures and images for: Multi-state occupancy models of foraging habitat use by the Hawaiian hoary bat (Lasiurus cinereus semotus)
Source: PLoS One. 2018 Oct 31;13(10):e0205150. doi: 10.1371/journal.pone.0205150 (PMC6209161; doi:10.1371/journal.pone.0205150)

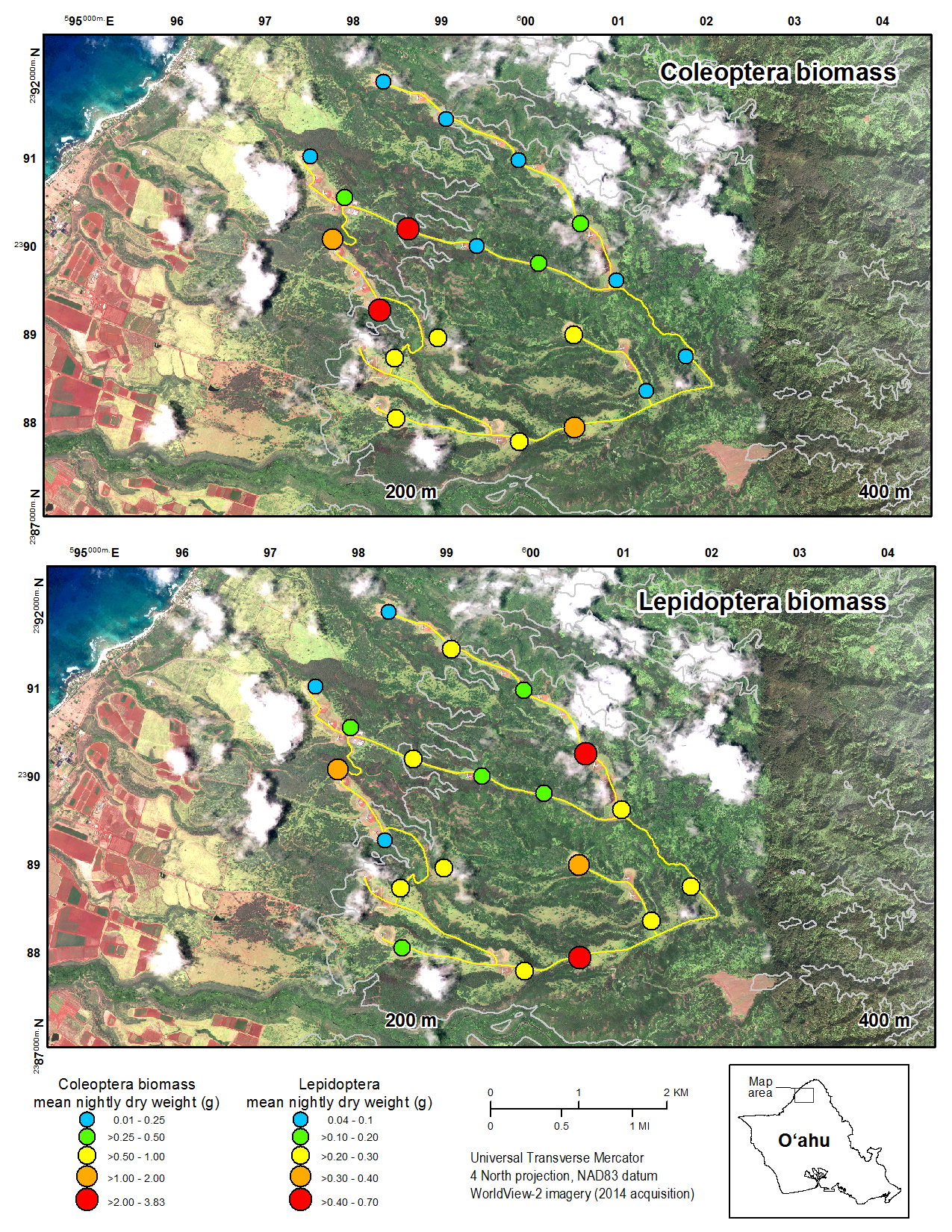


S1 Fig. Coleoptera and Lepidoptera biomass (mean nightly dry weight; grams) samples by site.

Supplement: S1 Fig — (DOCX) [file pone.0205150.s005.docx]
