# Supplementary material for: Multi-state occupancy models of foraging habitat use by the Hawaiian hoary bat (Lasiurus cinereus semotus)
Source: PLoS One. 2018 Oct 31;13(10):e0205150. doi: 10.1371/journal.pone.0205150 (PMC6209161; doi:10.1371/journal.pone.0205150)
Supplement: S2 Fig — (DOCX) [file pone.0205150.s006.docx]

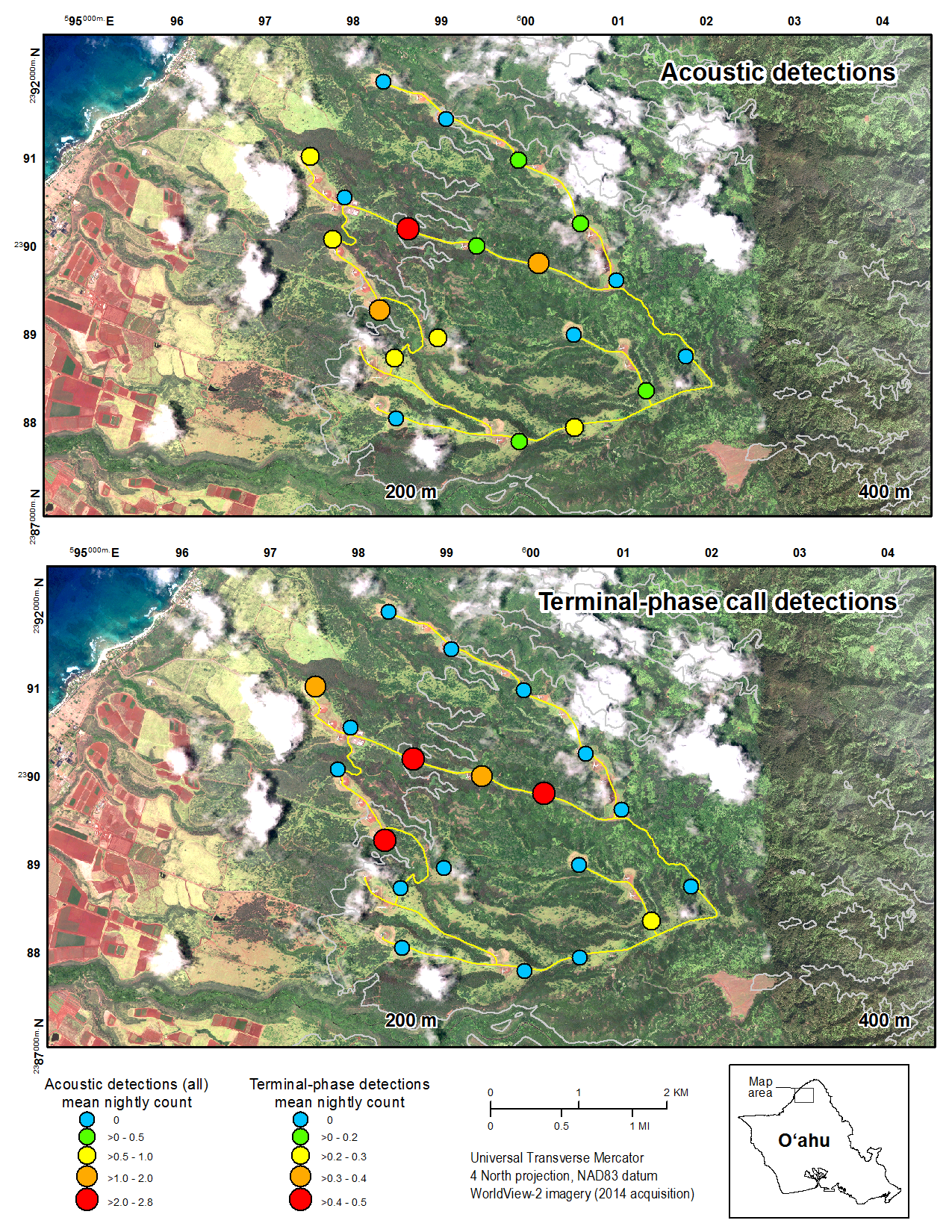


S2 Fig. Acoustic samples by site of Hawaiian hoary bats (*Lasiurus cinereus semotus*) and the subset identified as comprising feeding behavior (terminal-phase calls).
