# Supplementary material for: Multi-state occupancy models of foraging habitat use by the Hawaiian hoary bat (Lasiurus cinereus semotus)
Source: PLoS One. 2018 Oct 31;13(10):e0205150. doi: 10.1371/journal.pone.0205150 (PMC6209161; doi:10.1371/journal.pone.0205150)
Supplement: S3 Fig — (DOCX) [file pone.0205150.s007.docx]

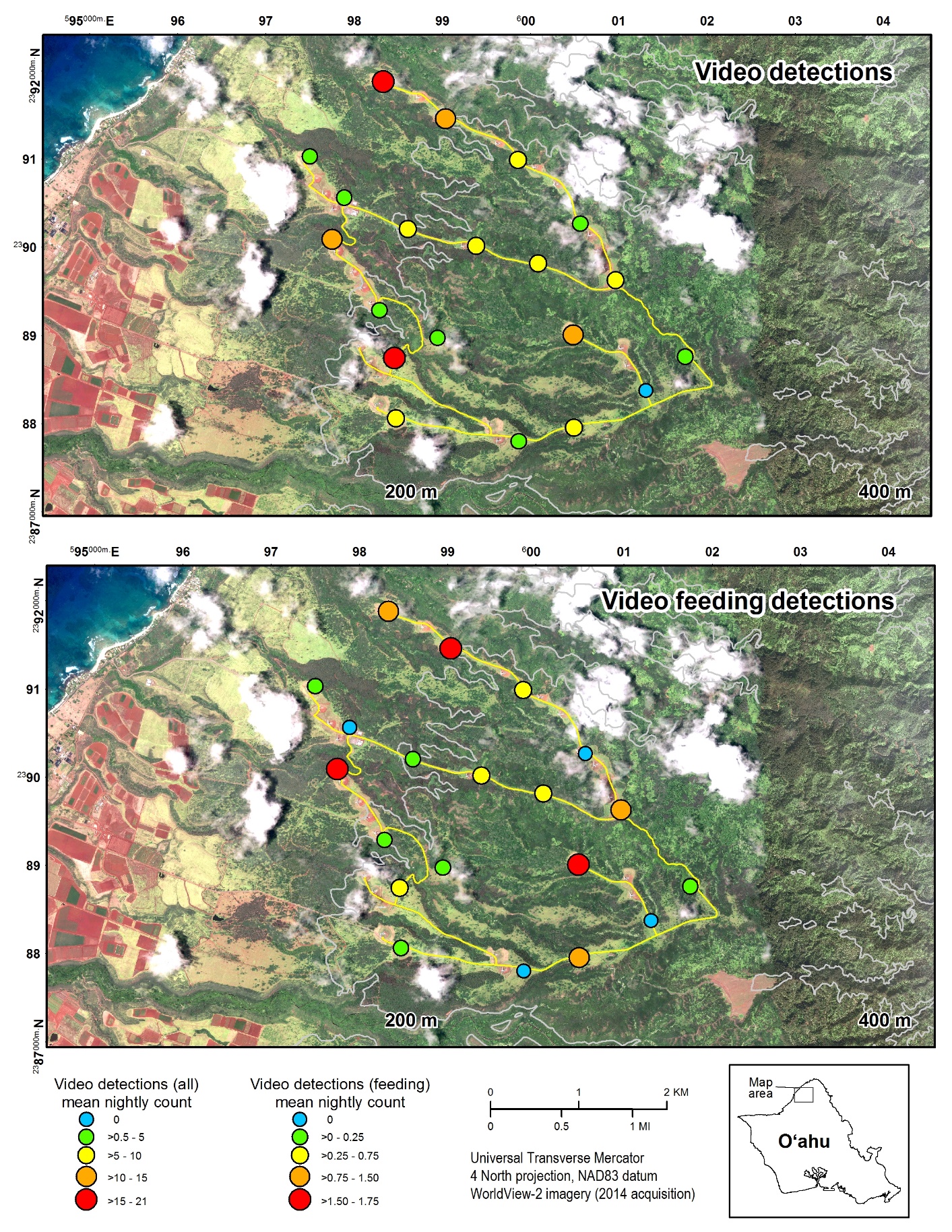


S3 Fig. Videographic detections by site of Hawaiian hoary bats (*Lasiurus cinereus semotus*) and the subset identified as comprising feeding behavior.
